# Practicing Tribal Sovereignty Through a Tribal Health Policy: Implementation of the Healthy Diné Nation Act on the Navajo Nation

**DOI:** 10.5888/pcd19.220106

**Published:** 2022-11-23

**Authors:** Regina Eddie, Caleigh Curley, Del Yazzie, Simental Francisco, Ramona Antone-Nez, Gloria Ann Begay, Priscilla R. Sanderson, Carmen George, Sonya Shin, Shirleen Jumbo-Rintila, Nicolette Teufel-Shone, Julie Baldwin, Hendrik “Dirk” de Heer

**Affiliations:** 1 School of Nursing, Northern Arizona University, Flagstaff, Arizona; 2 Department of Health Sciences, Northern Arizona University, Flagstaff, Arizona; 3 Mel and Enid Zuckerman College of Public Health, University of Arizona, Tucson, Arizona; 4 Navajo Epidemiology Center, Navajo Department of Health, Window Rock, Arizona; 5 Diné Food Sovereignty Alliance, Gallup, New Mexico; 6 Brigham and Women’s Hospital, Boston, Massachusetts; 7 Navajo Division of Community Development, Window Rock, Arizona

## Abstract

**Introduction:**

The Navajo Nation is a large sovereign tribal nation. After several years of grassroots efforts and overcoming an initial presidential veto, the Navajo Nation passed the Healthy Diné Nation Act (HDNA) in 2014 to promote healthy behaviors in Navajo communities. This was the first such policy in the US and in any sovereign tribal nation worldwide.

**Purpose and Objectives:**

The objective of this study was to describe the process, implementation, and evaluation of the HDNA passage and its 2020 reauthorization and the potential for using existing and tribal-specific data to inform tribal policy making.

**Intervention Approach:**

The HDNA included a 2% tax on unhealthy foods sold on the Navajo Nation and waived a 6% sales tax on healthy foods. HDNA-generated funds were allocated to 110 local communities for wellness projects. No funds were allocated for enforcement or compliance.

**Evaluation Methods:**

We assessed HDNA tax revenue and tax-funded wellness projects in 110 chapters over time, by region and community size. The food store environment was assessed for fidelity of HDNA implementation, price changes since pretax levels, and shopper behaviors. HDNA revenue was cross-matched with baseline nutrition behaviors and health status through a Navajo-specific Behavioral Risk Factor Surveillance System survey.

**Results:**

HDNA revenue decreased modestly annually, and 99% of revenue was disbursed to local chapters, mostly for the built recreational environment, education, equipment, and social events. Stores implemented the 2% tax accurately, and the food store environment improved modestly. Regions with high tax revenue also had high rates of diabetes, but not other chronic conditions. The HDNA was reauthorized in 2020.

**Implications for Public health:**

Sovereign tribal nations can drive their own health policy. Program evaluation can use existing data sources, tailored data collection efforts, and tribal-specific surveys to gain insight into feasibility, implementation, and impact.

**Summary**

**What is already known on this topic?**

Despite health disparities among American Indian and Native American populations, few sovereign tribal nations drive their own health policy. Unhealthy food taxes may reduce consumption of unhealthy foods, but this tax was implemented only recently in a single rural tribal nation.

**What is added by this report?**

We describe the passage, evaluation, and reauthorization of the Healthy Diné Nation Act (HDNA) of 2014, a 2% tax on unhealthy foods and a 6% sales tax waiver on healthy foods. Evaluation combined existing data with Navajo Nation–specific data.

**What are the implications for public health practice?**

Sovereign tribal nations with populations at high risk for negative health outcomes can shape their own health policy and use existing data sources and tribal-specific data to gain insight into intervention feasibility, implementation, and impact.

## Introduction

**Figure 1:**
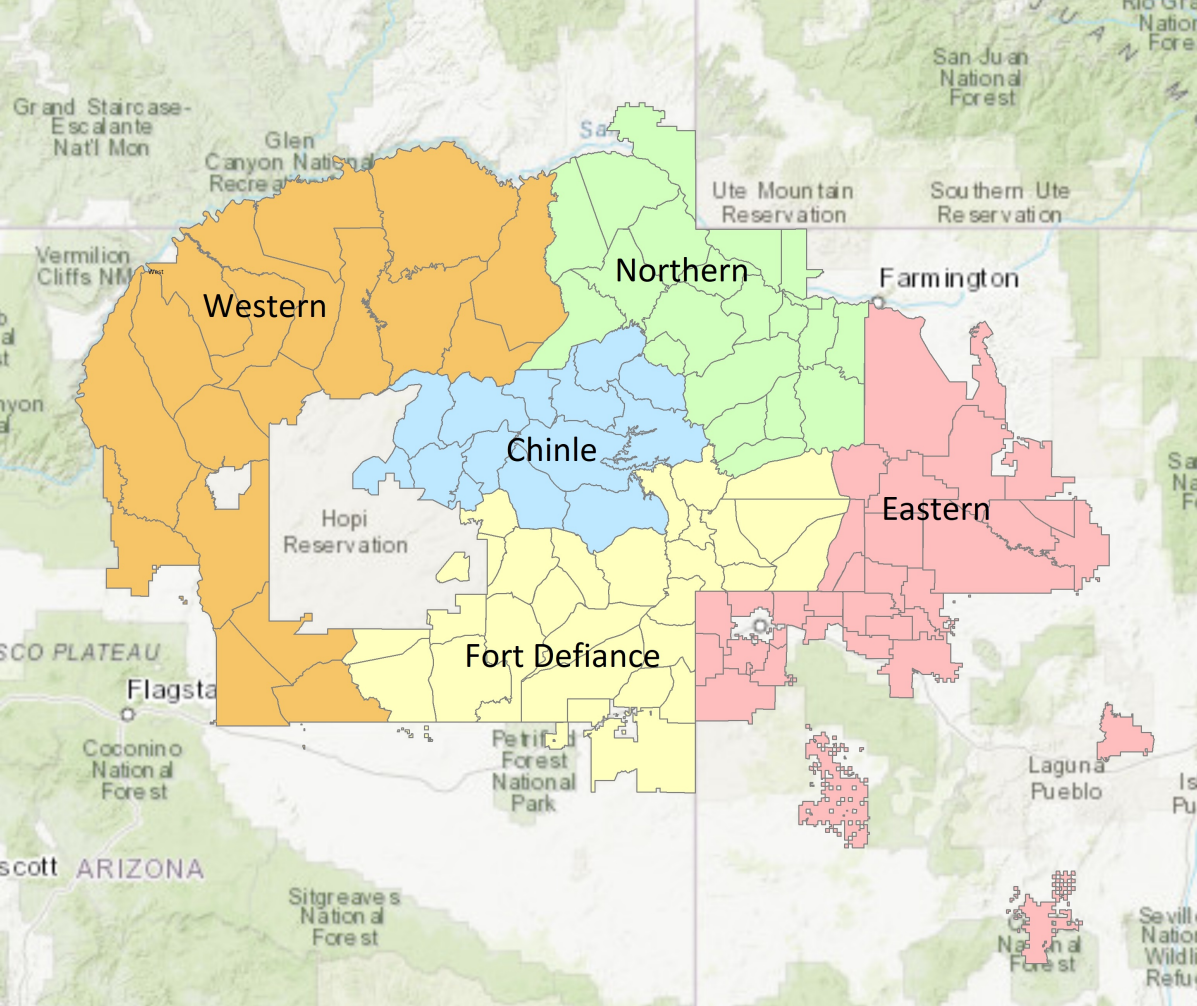
The 5 regions (also known as agencies) of the Navajo Nation: Chinle, Eastern, Fort Defiance, Northern, and Western. Sources: Esri, Esri China (Hong Kong), Esri Japan, Garmin, General Bathymetric Chart of the Oceans, GeoBase, GIS User Community, HERE Technologies, Institute Geographique National Increment P Corporation, Intermap Technologies, Kadaster International, Ministry of Economy, Trade and Industry, National Park Service, Natural Resources Canada, OpenStreetMap contributors, Ordnance Survey, United Nations Food and Agriculture Organization, and US Geological Survey.

The Navajo (Diné) people live in the largest American Indian reservation in the US. The reservation covers more than 27,000 square miles, extending into Arizona, Colorado, New Mexico, and Utah (Figure 1). The Navajo Nation consists of 5 regions (also known as agencies), and across these regions are small communities (also known as chapters) that have an average population of approximately 1,700 residents. Chapters function as the smallest level of government, where governance and planning are conducted by local elected officials. By population, Navajo is now estimated to be the largest American Indian tribe in the US; it has nearly 400,000 enrolled tribal members, of whom nearly half live on the Navajo reservation (1,2).

Like many American Indian tribes, the Navajo people experience higher rates of preventable nutrition-related diseases such as obesity, diabetes, heart disease, and cancer than the general US population (3,4). These disparities stem from the negative impacts of colonization; tribes have been forced to endure threats to their existence as a result of federal policies, forced assimilation, removal from tribal homelands, and relocation to reservation lands (5). Altogether, this history has been detrimental to traditional lifeways. Access to nutritious foods that were once produced by subsistence activities of farming, herding, hunting, and gathering have given way to greater reliance on processed foods high in fat, sugar, and salt (5). Poverty, unemployment, lack of transportation, and remote, rural geography with few grocery stores amplify the challenges to healthy food access (6).

Tribes are sovereign nations that are increasingly finding ways to challenge the health impacts of colonization by exerting their authority to improve, protect, and promote the health and welfare of their people and communities (7). However, these efforts are not without challenges. Many tribes lack reliable tribal-specific data that are needed to make innovative and forward-moving decisions (8). To help address this gap, tribes need to “govern the collection, ownership, and application of their own data” (9). Furthermore, there is a need for tribes to engage in more public health activities through tribal policy and implementation (7). A growing body of evidence recommends broader strategies, including policy-change initiatives to improve access to healthy foods (10,11). Taxing unhealthy foods and beverages along with healthy food subsidies is an example of a policy change that targets broad food environments.

The Navajo Nation has taken steps to assert its tribal sovereignty and self-determination to address health outcomes and restore *hózhó*, holistic health and wellness for its people, through its own data collection, policy development, and evaluation initiatives (6,12). For example, the Navajo Epidemiology Center, responsible for managing Navajo public health data, developed and implemented its own tribal health survey, the Navajo Nation Health Survey (NNHS), a tribal-specific Behavioral Risk Factor Surveillance System (BRFSS) survey (3). This survey adapted the rigorous BRFSS methodology, but was translated, conducted in person, and adapted to the setting and culture of the Navajo people. Such data can be valuable for monitoring and evaluating health trends, and planning, implementing, and evaluating health interventions relevant to a specific group or environment.

Along with building its tribal data capacity, the Navajo Nation is the only location in the US to use its authority to create a historic tax policy, the Healthy Diné Nation Act (HDNA) of 2014, which introduced a 2% tax on unhealthy foods and waived a 6% sales tax on fruit, vegetables, and water (13,14). Unhealthy foods include sweetened beverages and prepackaged snacks that have been stripped of essential nutrients and are high in salt, saturated fat, and sugar. Some examples of tax-eligible items include sugar-sweetened beverages, candy, chips, and sweetened baked goods (14,15). A growing number of countries and US cities have implemented higher taxes on unhealthy food products and sugar-sweetened beverages (16–24). The higher taxes have been associated with reduced consumption of unhealthy food and beverage products and increased consumption of water (16–24). Reducing sugar-sweetened beverage consumption by 10% to 20% can result in a 1.8% to 3.4% decline in new cases of diabetes, 0.5% to 1% in coronary heart disease cases, and 0.5% to 0.9% in myocardial infarctions (25). To date, no peer-reviewed literature has summarized the development or evaluation of an unhealthy food tax or similar legislation in a tribal nation with a population at high risk for preventable nutrition-related conditions. This article aims to address this gap.

## Purpose and Objectives

This article describes the process and implementation of the passage and reauthorization of the HDNA, and the potential for use of existing and tribal-specific data in program evaluation to inform tribal policy making. Evaluation data included 1) tracking tax revenue over time and by region, 2) summarizing HDNA-funded wellness projects implemented by chapters, 3) assessing implementation fidelity in Navajo Nation stores, 4) assessing changes in food pricing from pre-HDNA levels through observations, and 5) linking HDNA tax revenue by region to Navajo-specific health behaviors and outcomes to test whether higher tax revenue correlates with higher diabetes rates or less healthy dietary intake (Table 1). Insights from our findings can provide valuable information to other tribal populations considering similar legislation and tribal sovereignty efforts.

**Table 1:** Data Sources Used for Assessment of Implementation and Impact of the Healthy Diné Nation Act (HDNA) of 2014

Type	Data Source	Findings	Notes
**Tax revenue**			
• Over time (each fiscal year) • By Navajo Nation region	Navajo Office of the Tax Commission 2015–2019	Allocation averaged $1.8 million per year and decreased 3% annually without decrease in overall Navajo Nation retail tax decrease.	Cross-matched with Navajo Nation Division of Community Development data set of allocations to 100% match amounts.
**Wellness projects**			
• Over time • By Navajo Nation region • By community size	Navajo Nation Division of Community Development 2015–2019	More than 99% of funds were allocated to each of 110 chapters, primarily for built recreational environment, exercise equipment, instruction, and social events.	Allocations can be generated for each chapter.
**Fidelity of stores in implementing taxes**			
• 2% HDNA tax • 6% Waiver	Store purchases across Navajo Nation stores in 2019	Fidelity of 2% tax was 87%, but much lower (55.3%) for 6% waiver.	No funds for compliance or enforcement in the legislation.
**Food price changes**			
• Healthy and unhealthy Item purchases and observations from pre-tax levels.	Epi-AID survey in 2013 by Centers for Disease Control and Prevention and Navajo Epidemiology Center repeated in 2019.	Modest reductions in cost of fruit and increase in promotion of healthy foods. No changes in food availability.	Use of validated instruments such as Nutrition Environment Measurement Survey.
**Shopper and community surveys**			
• Surveys in 2017 and 2019 • Shopping habits, HDNA awareness	Shopper survey with 274, 236, and 332 participants at stores and communities on and around Navajo Nation	Awareness of HDNA, but not waiver, was high. Water purchases increased. Purchases of sugar-sweetened beverages decreased but remained high. HDNA support high.	—
**Nutrition behaviors**			
• Sugar-sweetened beverages, including fruit drinks • Fruits and Vegetables	Navajo Nation Health Survey (Navajo BRFSS)	Highest fruit and vegetable intake in region with lowest HDNA revenue, but not intake of sugar-sweetened beverages.	Cross-matched with HDNA tax revenue; follow-up to be collected in 2022
**Health outcomes**			
• Diabetes • Body mass index	Navajo Nation Health Survey (Navajo BRFSS)	Highest diabetes rates in region with highest HDNA tax revenue, but not body mass index.	Cross-matched with HDNA tax revenue; follow-up to be collected in 2022

Abbreviation: BRFSS, Behavioral Risk Factor Surveillance System.

## Intervention Approach

### Tribal policy-making structure

Similar to the US government structure, the Navajo Nation consists of 3 branches — executive, judicial, and legislative. Elected by the Navajo people, the president and vice president represent the executive branch and serve a 4-year term. The judicial branch consists of the chief of justice, who is appointed by the president. Finally, the 24 members of the Navajo Nation Council are elected to serve a 4-year term by registered voters in the 110 chapters. The Navajo Nation Council is responsible for enacting laws to protect the health and well-being of its communities (26). The intricate tribal legislation process involves sponsorship of legislation by a council delegate, intake and review of legislation, and assignment of legislation to a standing committee; it ends with a vote by the full Navajo Nation Council. If the majority of the Navajo Nation Council votes in favor of the legislation, it is then referred to the president to become law (26).

### Laying the groundwork for the HDNA

Established by Congress in 1997, the Indian Health Service’s Special Diabetes Program for Indians has funded community-based interventions to treat and prevent type 2 diabetes on the Navajo Nation (27). Despite these efforts, type 2 diabetes is a persistent public health problem. In 2011, the Navajo Area Indian Health Service, the regional agency responsible for providing federal health care services, shifted its approach to engage local community input to develop and implement community-based interventions. This reframing led to community mobilization efforts in which health champions were recruited across the 8 Indian Health Service units located on or near the Navajo Nation (Gloria Begay, Executive Director, Dine Food Sovereignty Alliance, personal communication [telephone], February 22, 2022).

The selected group of health champions included grassroots advocates with the Diné Community Advocacy Alliance (DCAA), who were key to the community movement and helped to lay the groundwork for the HDNA (28). DCAA and partners from the Navajo Area Indian Health Service and other health entities received training on how to be effective health advocates. They studied health data provided by the Navajo Area Indian Health Service and researched evidence-based solutions to decrease rates of type 2 diabetes in their community. They found various studies that implemented a taxation system on sugar-sweetened beverages and decided this would be a favorable solution system for their community. Not only would the taxation system promote healthy eating, but it would also allow communities to use their allocated tax revenue to develop their own chapter wellness projects.

### Intervention

The community collaboration led to the design of the HDNA, a taxation system that would require consumers to pay an additional 2% tax for unhealthy foods and would waive the 6% sales tax on healthy foods such as fresh produce, water, and nuts (12,13,15). Of all HDNA revenue generated, 20% would be set aside for the Permanent Trust Fund, the Veterans Trust Fund, and other related funds, and 80% would be allocated directly to all 110 chapters through their respective agency in a 50/50 formula: 50% allocated to all chapters and 50% distributed according to community size (ie, chapter voter registration) (14). Local chapters may use funds to develop and implement community wellness projects that address local health priorities and needs, which uniquely align with tribal government structures emphasizing local decision making and community support.

### HDNA and tribal decision-making process

**Figure 2:**
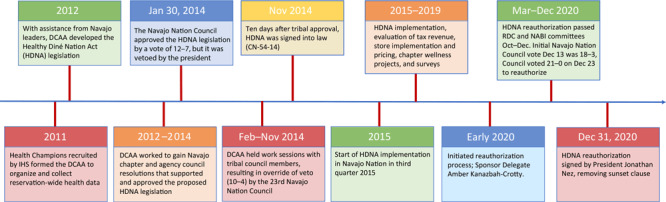
Timeline for implementation, evaluation, and reauthorization Healthy Diné Nation Act of 2014. Abbreviations: DCAA, Diné Community Advocacy Alliance; IHS, Indian Health Service; RDC, Resources and Development Committee: NABI, Nabikiya Committee.

HDNA community collaboration, passage, and reauthorization took place from 2012 through 2020 (Figure 2) (8). In 2012, the DCAA led the effort to secure Navajo Nation Council delegate sponsors to steer the HDNA through the Navajo legislative process. This effort also required gaining support from local communities. DCAA created educational materials and launched a reservation-wide education campaign on community health issues and introduced the HDNA as a proposed solution for these disparities. From 2012 through 2014 (2 years), DCAA presented information at 50 community chapter meetings, 20 standing committees, and 6 Navajo Nation Council meetings.

**Table:** 

Date	Activity
2011	Health champions recruited by IHS formed the Diné Community Advocacy Alliance (DCAA) to organize and collect reservation-wide health data.
2012	With assistance from Navajo leaders, DCAA developed the Healthy Diné Nation Act (HDNA) legislation.
2012–2014	DCAA worked to gain Navajo chapter and agency council resolutions that supported and approved the proposed HDNA legislation.
Jan 30, 2014	The Navajo Nation Council approved the HDNA legislation by a vote of 12 to 7, but it was vetoed by the president.
Feb-Nov 2014	DCAA held work sessions with tribal council members, resulting in override of veto (10 to 4) by the 23rd Navajo Nation Council.
Nov 2014	10 days after tribal approval, HDNA was signed into law (CN-54-14).
2015	Start of HDNA implementation in the Navajo Nation in third quarter 2015.
2015–2019	HDNA implementation, evaluation of tax revenue, store implementation and pricing, chapter wellness projects, and surveys.
Early 2020	Initiated reauthorization process; Sponsor Delegate Amber Kanazbah-Crotty.
Mar–Dec 2020	HDNA reauthorization passed the Resources and Development Committee (RDC) and the Nabikiya (NABI) Committee during October–December. Initial Navajo Nation Council vote December 13 was 18 to 3; Navajo Nation Council voted 21 to 0 on Dec 23 to reauthorize.
Dec 31, 2020	HDNA reauthorization signed by President Jonathan Nez, removing sunset clause.

By 2014, DCAA had gained 45 chapter resolutions that supported and approved the HDNA legislation. Additionally, 4 of 5 Navajo agency council resolutions approved the 2% tax legislation while one tabled it. DCAA met with all Navajo Nation Council standing committees and the executive branch several times to attain support and approval of the legislation. On January 30, 2014, the Navajo Nation Council approved the HDNA by a vote of 12 to 7; however, the Navajo Nation president vetoed it, requesting further clarification on HDNA implementation. DCAA immediately met in a series of work sessions for council presentations to the 4 Navajo standing committees and the full Navajo Nation Council to seek its override of the presidential veto.

On November 11, 2014, the HDNA was approved by the 23rd Council with a vote of 10 to 4, and 10 days later, the HDNA, CN-54–14 was signed into law by Navajo Nation President Ben Shelly. In the legislation, the Navajo Nation Council provided 5 years to plan and implement the HDNA (12). The development and implementation of the HDNA required collaboration, partnership, and support from various entities within the tribal government system. The Office of the Navajo Nation Tax Commission guided early implementation stages of how to collect HDNA tax revenue through a memorandum sent to retail establishments affected by the legislation. The legislation did not allocate funding for enforcement or monitoring of compliance. HDNA tax revenue collected from retail establishments (grocery and convenience stores, gas stations, restaurants, trading posts) located on the Navajo Nation are deposited into the Community Wellness Development Project Funds, to be appropriated pursuant to a funds management plan approved by the Budget and Finance Committee and administered by the Navajo Division of Community Development (DCD). The Navajo DCD is responsible for reviewing and approving community wellness projects and disbursing funds to the 110 chapters. Local chapter officials received training on the HDNA before receiving funds.

### HDNA reauthorization

The 2014 legislation included a sunset clause, which required that the 2% sales tax on unhealthy food and sugar-sweetened beverages cease at the end of 2020, unless reauthorized by legislation (13). To support the Navajo Nation Council in making an informed decision about HDNA reauthorization, a collaborative team evaluated the HDNA. The team included researchers, epidemiologists, and tribal leaders with joint expertise in program evaluation, research with tribal nations, Navajo culture, nutrition interventions, and health policy and included representatives from the Navajo Epidemiology Center, Navajo DCD, Office of the Navajo Nation Tax Commission, and researchers from several regional academic and tribal institutions.

Simultaneously, grassroot advocates sought Navajo Nation Council delegate sponsors to assist in the reauthorization of the HDNA. To inform Navajo Nation Council members, the evaluation team created and disseminated information packets that included a 20-slide Microsoft PowerPoint presentation describing tax revenue data, chapter wellness projects, store implementation and community surveys, and informational brochures summarizing which healthy foods were exempt from sales tax and which unhealthy foods were taxed an additional 2%. Additionally, reports on tax revenue and chapter wellness projects for each area represented by each council delegate were provided to educate tribal leaders and community members about the impact of the HDNA in their communities. Following votes in each legislative subcommittee and finally the full Navajo Nation Council on December 23, 2020, the Navajo Nation Council reauthorized the HDNA indefinitely by a vote of 21 to 0. The newly elected Navajo Nation president and vice president commended grassroots organizers, tribal leaders, and the Navajo Nation community for their efforts to promote healthy living through the HDNA initiative and traditional Navajo teachings known as *T’áá hwó’ajít’éego*, or self-reliance and self-determination (28).

## Evaluation Methods

Evaluation methods aimed to generate data relevant to the Navajo people and support the Navajo Nation Council in making an informed decision about HDNA reauthorization in 2020. First, data that were already collected were summarized; these were feasible and sustainable sources of information that provided a broad image of implementation. These included tax revenue and allocation of HDNA funds to local communities. Second, the food store environment was assessed because of its relevance to the feasibility of the legislation; we assessed pricing changes and the fidelity of implementation. Finally, several targeted surveys were conducted or analyzed, including baseline findings of the NNHS. All evaluation methods and dissemination materials were approved by the Navajo Nation Human Research Review Board (NNR17–284T).

### Tax revenue

Information on tax revenue was collected from the Office of the Navajo Nation Tax Commission. Reports provided information on total revenue generated each quarter of each Navajo Nation fiscal year since inception of the HDNA in 2015. Information on revenue allocation was categorized by Navajo Nation region and chapter, allowing for cross-checking of disbursements to communities by the Navajo DCD (14).

### HDNA-funded chapter wellness projects

Through the Navajo DCD, data were available on chapter wellness project proposals and allocations. Each year, each chapter received notice of their allocated amount by the Navajo DCD and was requested to submit a project proposal after community consultation. Instructions in the form of a memorandum provided examples of the types of projects detailed in the HDNA legislation (13). The proposal required a brief text description of planned activities (on average just over 50 words) and amount of funds requested for each activity (29). Data retrieved for evaluation included total amount of funds, year, chapter community, Navajo Nation region, and the full text of each proposal. Data were categorized by 3 independent coders into 9 categories (eg, built recreational environment, equipment, social events, instruction) and 43 subcategories (eg, basketball courts [built recreational environment], traditional foods classes [instruction]). Changes in types of community projects were tracked over time and by region. By combining the data with chapter community population (2), we could further assess differences by community size (29).

### Food store environment: HDNA implementation and pricing

Changes in food pricing and availability and fidelity of HDNA implementation in the food store environment were also assessed. Data obtained from a pre-HDNA survey of 83 stores served as a baseline. This assessment of the Navajo Nation food environment in 2013 was conducted by the Centers for Disease Control and Prevention and the Navajo Epidemiology Center (30). The 83 stores included 63 Navajo Nation stores (13 grocery stores and 50 convenience stores) and 20 border-town stores. In 2019, the survey was replicated in all 51 stores that were still operational. Of the 32 stores that were no longer operational, a computer-generated randomized algorithm was used to match 20 original stores to 20 new operational stores, based on type (convenience store/grocery store), location (on/off reservation), and region. The final 2019 sample consisted of 71 stores (51 original and 20 new matched stores) (31).

Changes in pricing and availability were assessed by using the Nutrition Environment Measurement Survey–Stores (NEMS–S) (32,33), a validated observational tool used to assess the nutrition environment in community food and retail outlets; it was also used in the baseline survey (30). Surveyors engaged in formal in-person training on the NEMS-S and gained verbal approval to conduct store assessments before data collection. Surveyors observed details such as signage, displays, and foods offered, and recorded pricing. The NEMS-S uses detailed standard protocols on everything from the time the store was visited to how to record pricing if items were not labeled (32,33). An example series of instructions is “Look for low-fat milk (skim or 1%). If available, mark ‘yes’. If not available, mark ‘no’ and look for 2% milk. Mark whether or not it is available. Look for the store brand. If available, mark ‘yes.’ Record the price of a quart and [a] half-gallon milk.”

Evaluation also included assessing the fidelity of HDNA implementation in stores. Because no funding was available for HDNA compliance or enforcement, fidelity of implementation depended on stores, which received a memorandum and guidance from the Office of the Navajo Tax Commission. In 2019, implementation fidelity was evaluated in 47 stores (33 convenience stores, 8 grocery stores, 6 trading posts) by purchasing 2 tax-eligible (unhealthy) items and 2 tax-exempt (healthy) items in each store and calculating the appropriate taxation level. Taxes on unhealthy items included state sales tax (0% in Arizona, New Mexico, Colorado, or 1.75% in Utah), Navajo Nation sales tax (6%), and the HDNA tax (2%) (34). Acceptable ranges were defined as within 0.5% above or below the appropriate taxation level. To calculate the percentage of stores that implemented the tax accurately, we divided the number of stores that implemented accurately by the total number of stores.

### Community and shopper surveys

To gain insight into individual shopping behaviors and awareness and support of HDNA among shoppers, biannual surveys were conducted in 2017 (July 2017 until January 2018) and 2019 (June–November) (35). These surveys used convenience samples in Navajo Nation communities or samples of shoppers consecutively leaving stores on the Navajo Nation, with sample sizes ranging from 235 to 332. The timing of store visits was randomized via computer-generated assignment based on weekday/weekend, beginning/end of month, and AM/PM times. A minimum of 2 store visits were made at each store to provide a broad sample of shopping behaviors (35). Questions included whether participants purchased fresh produce, water, or sugar-sweetened beverages and whether they were aware of and in support of the HDNA. The survey instrument was based on prior longitudinal store interventions, pilot tested at 3 store locations, and adjusted for word choice, reading level, and cultural appropriateness. Trends were examined over time to determine if purchasing patterns became healthier over time.

### Navajo Nation Health Survey

Finally, to gain insight into associations between tax revenue, nutrition behaviors, and health outcomes, we used the NNHS. The methodologic approach included selecting samples in 3 levels of census block groups and using Esri ArcGIS Explorer to superimpose an aerial map to select high-density and low-density cells. Structures were then randomly selected within these cells and approached, and adults in each household were randomly selected for participation. The total number of participants was 2,346, with a response rate of 39% (3). Survey data were collected in 2013, 2015, and 2016; unlike the national and state BRFSS, surveys were conducted in person to allow for translation in Navajo language and to address limitations in cell phone reception. Survey adaptations also considered sociocultural, language, and community contexts. The final NNHS consisted of 199 questions that took 90 to 120 minutes to administer and covered a broad range of health domains, including chronic diseases (eg, diabetes), body mass index (BMI), and nutrition, including intake of sugar-sweetened beverages and fruits and vegetables (3). NNHS health status and behavior estimates were adjusted to account for population age and sex distribution at the Navajo Nation regional level, allowing us to test whether regions with higher HDNA revenue (more unhealthy food purchases) reported higher diabetes rates, higher intake of sugar-sweetened beverages, and lower fruit and vegetable consumption.

### Data analysis

Data analysis was focused on generating relevant and comprehensible reports for the Navajo people and Navajo Nation Council. Tax revenue and chapter wellness projects were summarized in Excel (Microsoft Corporation) and cross-matched for accuracy of distribution allocations. Data on store implementation, pricing, and availability and NNHS nutrition and health status were collected in Excel and analyzed by using SPSS version 27.0 (IBM Corporation) to generate frequency distributions and descriptive statistics and test for trends over time. Regression analyses were used to test the association between HDNA revenue and health outcomes (diabetes and obesity) and nutrition intake. Covariates were included in multivariate models if they were significantly associated with outcome on bivariate analysis. Analyses for diabetes and obesity also adjusted for proportion of residents reporting daily intake of sugar-sweetened beverages. Esri ArcGis version 10.7.1 was used to generate graphic representations of the main findings.

## Results

### Tax revenue

From implementation of the HDNA in the fourth quarter of fiscal year 2015 through the fourth quarter of 2019, the gross HDNA tax revenue generated totaled $7.58 million, approximately $1.8 million per year and $13,000 per year for each of the 110 chapters. Revenue modestly but significantly declined over time by an average of approximately 3% per year (14).

### HDNA-funded chapter wellness projects

Of all HDNA revenue allocated to chapters, more than 99% ($4.61 million of $4.64 million available to 437 of 440 chapters) was successfully distributed; only 1 chapter each year did not submit a proposal on time (29). Chapters proposed about 3.5 types of activities per year (>1,300 activities in 2015–2018 alone) (29). The most common category chosen by chapters for their wellness projects was the built recreational environment, accounting for nearly 40% of all funds. The most common built environment subcategories were walking trails, basketball or volleyball courts, parks, and playgrounds. Next, about 15% of funds were allocated to exercise equipment, nutrition and fitness instruction, and social events. The types of projects chosen by chapters did not change significantly over time but did differ significantly by region. Regions with smaller allocations dedicated more funds to traditional, agricultural, and intergenerational projects and less to the built environment (29).

### Food store environment: HDNA implementation and pricing

Food store assessments indicated that fidelity of implementation was high for the 2% tax, with estimated fidelity of approximately 87% (41 of 47 stores) (34), comparable to other settings (36,37). However, the application of the 6% waiver was less consistent and closer to 50%. The price of fruits in Navajo stores decreased by 13% in 2019, compared with pre-HDNA prices, while pricing trends for vegetables and other healthy foods were inconsistent (31). Navajo convenience stores had greater availability of fresh vegetables and similar availability of fresh fruits compared with border-town convenience stores. Availability of traditional foods and health promotion signage also increased over time in Navajo stores.

### Community and shopper surveys

Among shoppers at Navajo stores, purchase of sugar-sweetened beverages was fairly common; however, from 2017 to 2019, the ratio of purchasing sugar-sweetened beverages to water improved significantly (35). A survey in 21 communities further found that levels of support for the HDNA (29) were moderate to high compared with research in other settings (38), with modestly greater support among participants with higher levels of education and income.

### Navajo Nation Health Survey

**Figure 3:**
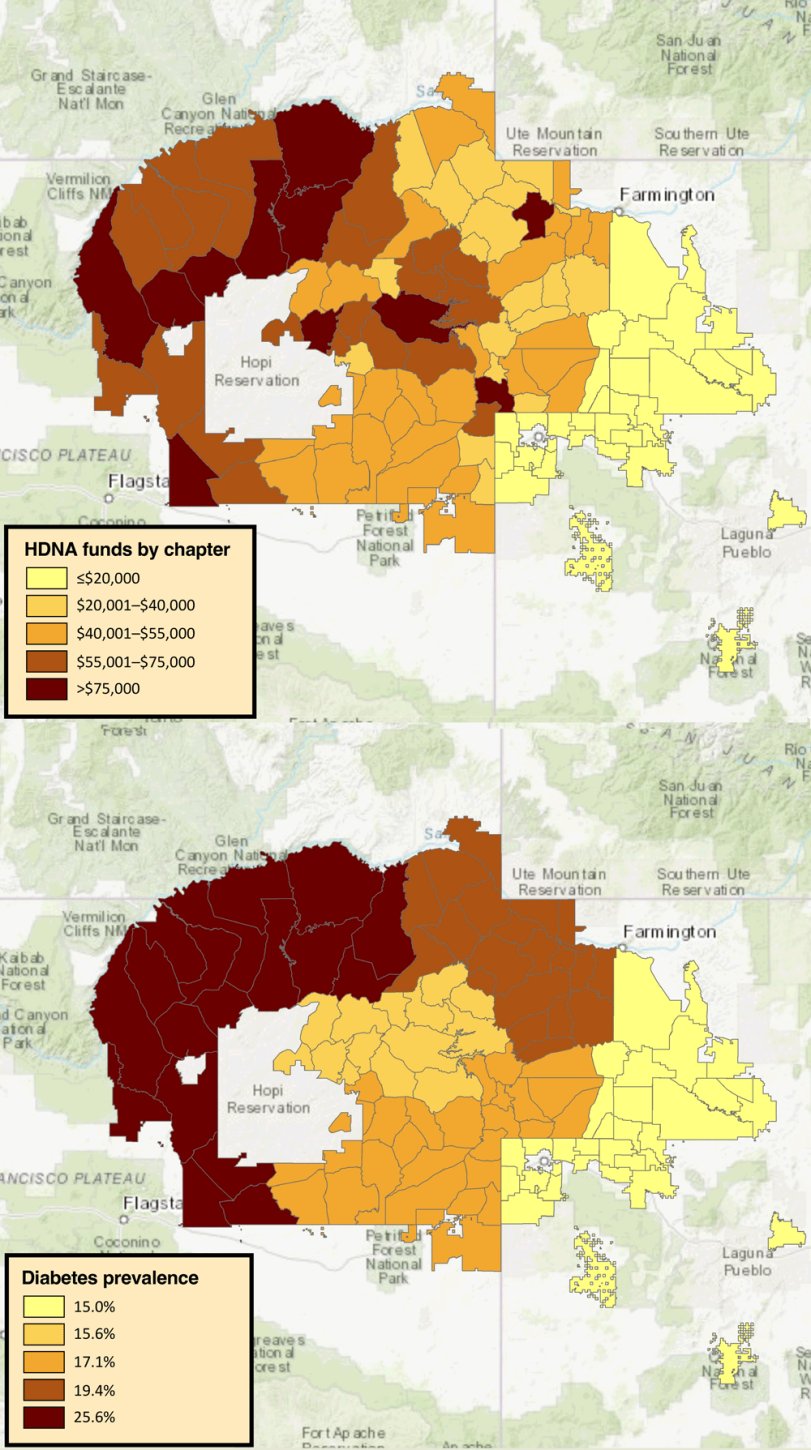
HDNA tax revenue, by chapter, and baseline diabetes prevalence, by region, on the Navajo Nation. Tax revenue was collected from fourth quarter of fiscal year 2015 through fourth quarter of fiscal year 2019. Baseline data collection of diabetes prevalence took place from 2013 through 2016. Abbreviation: HDNA, Healthy Diné Nation Act of 2014.

The region with the highest HDNA revenue ($2,133,199) was Western (Table 2); this region also had the highest mean baseline diabetes rate (25.6%) (Figure 3) and highest mean BMI (31.8 kg/m^2^). The area with the lowest HDNA tax revenue (Eastern, $408,414) had the lowest diabetes rate (15.0%) but not the lowest BMI. However, associations between HDNA revenue and diabetes and BMI were not significant (controlling for income and intake of sugar-sweetened beverages). Servings of fruits and vegetables (5.2 servings/day) and dark green vegetables (0.9 servings/day) were also the highest in the Eastern region, but so was daily intake of sugar-sweetened beverages (1.2 beverages per day), suggesting potential inconsistencies or underlying complexities.

**Table 2:** Revenue Generated by the Healthy Diné Nation Act of 2014 (HDNA), Baseline Diabetes Rate, Mean Body Mass Index, and Consumption of Selected Food and Beverage Items, by Navajo Nation Region^a^

Region	Total HDNA revenue, $	Baseline diabetes prevalence, %	Mean BMI, kg/m^2^	Consumption, mean no. per day				
				Sugar-sweetened beverages	Fruit drinks	Fruit and vegetable servings	Dark green vegetable servings	Fruit servings
Chinle	1,611,141	15.6	29.4	1.1	0.9	4.7	0.9	1.1
Eastern	408,414	15.0	30.6	1.2	0.8	5.2	0.9	1.4
Ft. Defiance	2,084,743	17.1	29.2	0.7	0.4	3.5	0.7	0.9
Northern	1,340,690	19.4	30.0	0.8	0.9	3.5	0.6	1.0
Western	2,133,199	25.6	31.8	0.8	0.8	4.8	0.7	1.4

Abbreviation: BMI, body mass index.

^a^ Data on HDNA revenue were collected from fourth quarter of fiscal year 2015 through the fourth quarter of fiscal year 2019. Data on diabetes, BMI, and nutrition were collected from 2013 through 2016.

**Table:** 

Category of HDNA funding received	No. of chapters
$20,000 or less	31
$20,001 to $40,000	19
$40,001 to $55,000	28
$55,001 to $75,000	21
More than $75,000	11

## Implications for Public Health

Tribal sovereignty ensures that any decisions about the tribes are made with tribal participation, consent, and meaningful culturally relevant data (7–9). The historical decline in tribal populations along with external threats to tribal sovereignty over land, food systems, and natural resources have long-term consequences to the health and longevity of tribal communities (6). In this study, we demonstrated the capacity of tribal nations to design their own public health intervention through a tribal policy-making process. The Navajo Nation’s HDNA project is an example of a community-based initiative, where local community members, tribal health and academic partners, along with existing infrastructure, such as tribal government and the Indian Health Service, were mobilized to initiate community and tribal-level health policy changes.

Community engagement honors local voices and ensures that they are heard and is critical for initiating community-level health changes (39). By mobilizing the community, insights on the breadth and depth of community health problems were gathered, local priorities were identified, and community-based health planning began. Tribes have their own legal system, usually an elected tribal council or legislative body, that can address needs of the community through policy development (7).

This study also confirms the use and value of existing data sources, such as the NNHS, for gaining insight into the feasibility, implementation, and impact of a system-wide intervention. The NNHS collects tribally specific data highly relevant to the Navajo people. The HDNA and NNHS are 2 unique initiatives developed in parallel, each playing a vital role in advancing the health of the Navajo people. The HDNA is a unique tribal health policy developed by and for the Navajo people in response to diseases affecting their communities. The NNHS rigorously measures health outcomes and behaviors in a way that is relevant and culturally appropriate for the Navajo people.

This article examined the relationship between HDNA tax revenue and NNHS data by region. Such data can be valuable in determining what is working or what needs to improve. For instance, if areas with higher HDNA tax revenue also have the highest diabetes rates, these areas may be prioritized for intervention. On the other hand, findings that suggest regions where less HDNA revenue corresponds to healthy dietary behaviors (purchase more fruits and vegetables) are encouraging. At minimum, these data are preferable to policy makers compared with multiple, broad-based statewide surveys that cover many different populations and tribal nations (eg, Arizona and New Mexico combined include >40 tribes). Having ownership of such information further aligns with a growing trend for tribal data governance, where data are produced and owned by tribes, and a term coined by some indigenous scholars: “indigenous data sovereignty” (8).

This analysis has at least one limitation. First, it focused on a single tribal population. As such, our findings may not necessarily be transferable to other tribes and indigenous societies. Each population has its own unique characteristics, political structures, and geographic context. Despite this limitation, our work has relevance for tribal nations and other indigenous societies that are considering tribal health policies to promote better nutrition behaviors.
